# What is herd health management? A narrative review

**DOI:** 10.1186/s13028-025-00849-y

**Published:** 2026-06-19

**Authors:** Esben Østergaard Eriksen, Ken Steen Pedersen, Jens Peter Nielsen

**Affiliations:** Department of Veterinary and Animal Sciences, Faculty of Health and Medical Sciences, University of Copenhagen, 1870 Frederiksberg C, Denmark

**Keywords:** Biosecurity, Cattle, Herd health, Management, Pig

## Abstract

**Background:**

This narrative literature review proposes an improved definition of herd health management and related terms. The role of veterinarians has shifted to a population-level oriented approach which may be denoted herd health management. Herd health management and related terms are used inconsistently in the scientific literature and theoretical deliberations around its practice are lacking.

**Results:**

Herd health management was defined as a continuous, cyclic process dealing with planning, implementing, and evaluating decisions aiming to promote herd health, i.e., normal biological function, including homeostasis and the absence of disease among the animals populating a given farm, to achieve the producer’s goals regarding productivity, animal health, and externalities.

**Conclusions:**

The paper proposes a theoretical framework describing how herd health management is practiced. Herd health management includes decisions at both strategic, tactical and operational level. The steps involved in tactical decisions are an evaluation of the current situation, defining goals, identifying risks and planning new practices to be implemented and a way to monitor indicators of success.

## Background

In many countries, an intensification of livestock production took place in the decades after the Second World War. For instance, in Danish pig production, antimicrobial supplements in the diet, climate-controlled pens, enhanced biosecurity, and more knowledge about feed paved the way for a noticeably lower age at which the producers separated the piglets from the sow [1]. In the 1970s, the common weaning age dropped from 7 to 10 weeks of age to approximately 4 weeks of age [1–3]. This intensification entailed the introduction of herd health management in pig production according to principles first developed in dairy production (see [4] for a historical overview). The role of veterinarians shifted from single animal-oriented and treatment-oriented to a population-level oriented and prevention-focused approach (see [5, 6] for comprehensive introductions). This development was reflected in the establishment of European College of Bovine Health Management and European College of Porcine Health Management in 2003 and 2004. In other words, today veterinarians provide herd health management consultancy, but what exactly is herd health management?

In this narrative review, we first propose definitions of some central terms in the field of herd health management. We then proceed by proposing a theoretical framework for herd health management and elaborating in detail on each step in the proposed framework.

## Methods

This narrative review was based on a search in Web of Science (http://www-webofscience.com) using the term “herd health” with no restrictions on the time of publication. In addition, our own archives were used as a source of additional information.

While the proposed framework and theoretical concepts is based on literature on production animal practice in general and mainly focused on cattle and pig production, we have selected examples referring to actual practices, diseases, etc. only from pig production, since we are most familiar with this production.

## Results and discussion

### Definitions herd health management and related terms

Various descriptions and definitions of herd health management and related terms have been proposed in the scientific literature. Below follows a selection of definitions.

Blood et al. gave one of the first definitions in 1978. They defined a **health** **program** *“as a planned and coordinated approach to achieving and maintaining optimal health and productive efficiency of livestock”* [7].

A year later, Blood defined **herd health** as “*a coordinated program of preventive measures to maintain the health of the herd”* [8].

Lissemore [9] cited Schnurrenberger [10] and defined the goal of a **herd health program** to be *“the maintenance of animal health and production at the most efficient level that will provide maximum economic returns to the farmer”* [9].

Lourens and Coubrough defined a **herd health program** to be “a planned and co-ordinated approach which aims at achieving and maintaining optimal herd health, production and reproduction, the implementation of which is governed by sound economic principles” [11].

In 2001, Radostits adhered to Blood’s definition and elaborated further that **herd health programs** “vary from simple systems, in which the veterinarian visits the herd on a regular basis to examine animals and their performance and to make recommendations for the control of disease and improvement of production, to intensive programs, in which the veterinarian—with the assistance of other agricultural science specialists—makes detailed recommendations about the daily management of the animal health production program” [5].

LeBlanc et al. described **health management** as *“an integrated*,* holistic*,* proactive*,* databased*,* and economically framed approach to prevention of disease and enhancement of performance”*; and defined it as *“the promotion of health*,* improvement of productivity*,* and prevention of disease in animals within the economic framework of the owner and industry*,* while recognizing animal welfare*,* food safety*,* public health*,* and environmental sustainability”* [6].

Hall and Wapenaar defined **herd health and production management** as *“regular scheduled farm visits that go beyond the ‘one-off’ tasks such as pregnancy diagnosis*,* castrations and dehorning. The purpose being to prevent disease and/or improve animal health and production by introducing long-term strategies focusing on the herd as a whole.”* [12].

Svensson et al. elaborated upon **veterinary herd health programs** and defined their aim *“to reduce the incidence of clinical and subclinical disease”*, and furthermore *“to give advice on how to reduce the risk of disease*,* how to optimize production*,* and later to follow up on the preventive measures applied”* [13].

Skjølstrup et al. adhered to the definition suggested by LeBlanc et al. [6], and added that **herd health management** approaches varies between countries; however, the **veterinary herd health consultancy** *“in focus in this review article is defined by a continuous collaboration between a farmer and the same veterinarian*,* with regular herd visits and a focus on herd health and production”* [14].

Gertzell et al. defined **veterinary herd health management** as a ***“****concept based on regular herd visits*,* taking all aspects of the production*,* such as anamnesis*,* production figures and clinical observations*,* but also e.g. the environment*,* management*,* feed and biosecurity*,* into account”* [15].

From the quotes above, it can be deduced that the literature contains a plethora of definitions of herd health management and related terms. It is not a comprehensive overview, but this selection of definitions is adequate to demonstrate that the field lacks common terminology. Additionally, the theoretical framework for practicing and studying herd health management was poorly described in the literature.

### Decomposing herd–health–management

What is a “herd”? What is “health”? What is “management”? Defining these terms separately may be helpful in defining herd health management.

A **herd** is a group of animals, typically cattle, pigs, poultry or related wild animals, that live together as a consequence of a natural behavioural pattern or because it is imposed by humans [16]. In the present context, a herd is a group of livestock living on the same farm. This definition makes it clear that working with a herd means working at the population level.

Animal **health** is difficult to define [17]. A review of 500 veterinary textbooks identified 39 heterogeneous definitions of health. Definitions of animal health emphasize one or more of the following five domains: normality, biological function, homeostasis, physical and psychological well-being, and productivity, including reproduction [17]. A doctoral thesis [18], aimed to present the various definitions of animal health. This concluded in somewhat agreement with [17] that health has been viewed as (1) biological function, more specifically normal biological function, biological homeostasis, or productivity, including reproduction; (2) mental and physical control; and (3) the ability to reach goals [18].

We did not include productivity in the definition of herd health. First, improving health is often viewed as a means to improve productivity, so it is wise to keep the terms separate. More importantly, the inclusion of productivity in animal health definitions is an old idea [18], but today it is rarely done [17, 18], and it seems to rely on a logical fallacy and an anthropocentric viewpoint [18]. Poor health will often impair productivity, e.g., disease hampers growth. Thus, animals suddenly displaying low productivity are likely to have poor health. Because of this association, it is tempting to define health based on productivity measures. Yet, as noted by Lerner, there are well documented cases of animals kept in production systems, where high productivity have direct and severe negative impacts on their health [18], e.g., high milk yield in cattle [19].

The noun **“management”** has been defined in different ways related to the process of dealing with or controlling things or people. In an agricultural context, farm management science, **“management”**, “decisions-making,” and “problem solving” have been used interchangeably in the literature to describe the management practices of farmers. The three concepts are overlapping in many ways and have evolved from common roots [20]. The study of general farm management goes back to the 1950s [20]. Theoretical frameworks/models of the management process are well-developed in this field and have been reviewed (see [20]). **Farm management** relates to all aspects of running a farm and can be defined as a continuous process dealing with planning, implementing, and evaluating decisions regarding production, finance, marketing, and human resources at a strategic, tactical, and operational level [20].

### Concise definitions of herd health management and related terms

Reviewing the literature helped identify terms lacking definitions in the field of herd health management. Concise definitions are therefore proposed inspired by previous suggestions of definitions of herd health management and related terms (see examples above), and the definitions of herd, health, and management. The definitions are listed below.

**Herd health** is the presence of normal biological function, including homeostasis and the absence of disease, among the animals populating a given farm.

**Herd health management** is a continuous, cyclic process dealing with planning, implementing, and evaluating decisions aiming to promote herd health, i.e., normal biological function, including homeostasis and the absence of disease among the animals populating a given farm, to achieve the producer’s goals regarding productivity, animal health, and negative externalities.

It is unclear what distinguishes **veterinary herd health management** from herd health management, and it is recommended to avoid this term. However, since the specific context and biological conditions differ among animal species it is relevant to distinguish between e.g. porcine health management and bovine health management.

**Herd health consultancy** is the task of providing expert assessments and advice to a livestock producer on how to promote herd health.

**Veterinary herd health consultancy** is the same as *herd health consultancy*, however, specifying that the advice is delivered by a veterinarian rather than other types of consultants.

A **herd health management program** is a more or less flexible outline for the delivery of herd health consultancy offered to livestock producers by a veterinarian or consultant (independent or employed by the livestock production enterprise), a veterinary practice, a consultancy company, an association, a retailer, a research institution, or the like; it is a scheme defining items such as themes and services to be covered in the consultancy, intervals of herd visits, specific data to be collected and evaluated, etc.

A **herd health management concept** is a flexible draft of how a certain herd health problem should be assessed and/or solved; it is typically based on evidence and/or expert opinions; and it typically consists of fixed operating procedures or a set of tools guiding the decisions regarding the specific herd health problem.

### Building a theoretical framework

A theoretical framework for herd health management is proposed below. Descriptions of herd health management were available in the scientific literature (see selected examples above) and frameworks proposed in closely related fields provided important inspiration. Below follows a description of how these ideas were modified to propose a framework for herd health management. For simplicity, the present and the following sub-paragraph will use the term “veterinarian”, when the role of both “herd health consultants” and “veterinary herd health consultants” is discussed.

As evident from our previously listed definitions, and as previously concluded [21], researchers agree that (1) herd health management follows a cyclic pattern of planning, implementation (also termed ”execution”), and evaluation (also termed ”control”); and (2) veterinarians contribute to the planning and evaluation at regular herd visits.

**Fig. 1:**
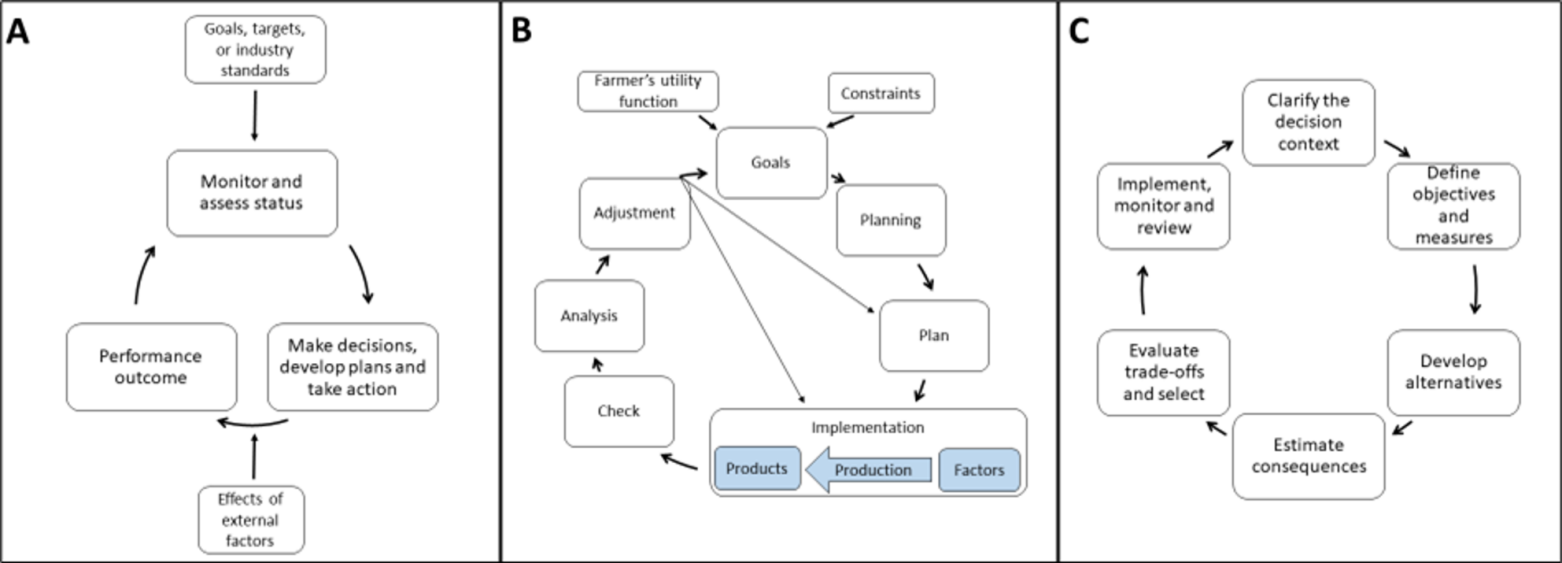
Three models of management cycles (Redrawn from Gregory et al. [23]; Kristensen et al. [24]; Radostits [5]. LeBlanc et al. [6] presented a figure identical to **A**)

A simple model of herd health management adhering to the above was proposed in 2001 [5]. The model is displayed in Fig. 1, Panel A, outlining a cyclic process where decisions continually seek to close gaps between goals and actual performance [5]. This was a good foundation to build a framework, but it lacked specification in several instances.

### A model of the planning–implementation–evaluation horizon

Decisions can be characterized by their level (strategic, tactical, or operational), primary function (planning, implementation, or control), and how structured the decision-making process is (from structured to unstructured) [20]. A model suggesting the structure for the ever-ongoing cyclic process of planning, implementation, and evaluation adequately described dairy producers’ feed management [22]. The dairy producers made major planning and evaluation decisions on a regular basis. Frequent, but smaller and more operational implementation and evaluation decisions were made in-between the planning decisions [22]. This will likely also capture the typical planning-implementation-control horizon of herd health management: Tactical planning decisions are made at regular herd visits, guided by the veterinarians’ advice. In between herd visits, the livestock producer and the herd personnel work on the implementation of the plan and make smaller operational decisions in accordance with the plan. Evaluation is carried out at the next herd visit.

### Models of the tactical planning decisions

It was pursued to embed a more detailed model for the tactical decisions made at the regular herd visits in the framework.

Environmental management shares the following features with herd health management: Multiple stakeholders (the veterinarian, the producer, and the herd personnel) make decisions based on different values and goals, and this is aided by the application of science-based knowledge in a specific context with a level of uncertainty in the prediction of the outcomes [23]. A well-described and widely applied framework had been developed in this field [23].

The model from environmental management science [23] resonated very well with a model proposed for herd (production) management [24]. The similarity can be recognized in Fig. 1, where both frameworks are displayed in panels B and C: Both models describe cyclic, continuous processes, and there is correspondence between the different stages of decisions that the two models include. Variations of these decision stages are also described in the seven different models of decision-making processes in farm management [20].

### A theoretical framework for herd health management

**Fig. 2:**
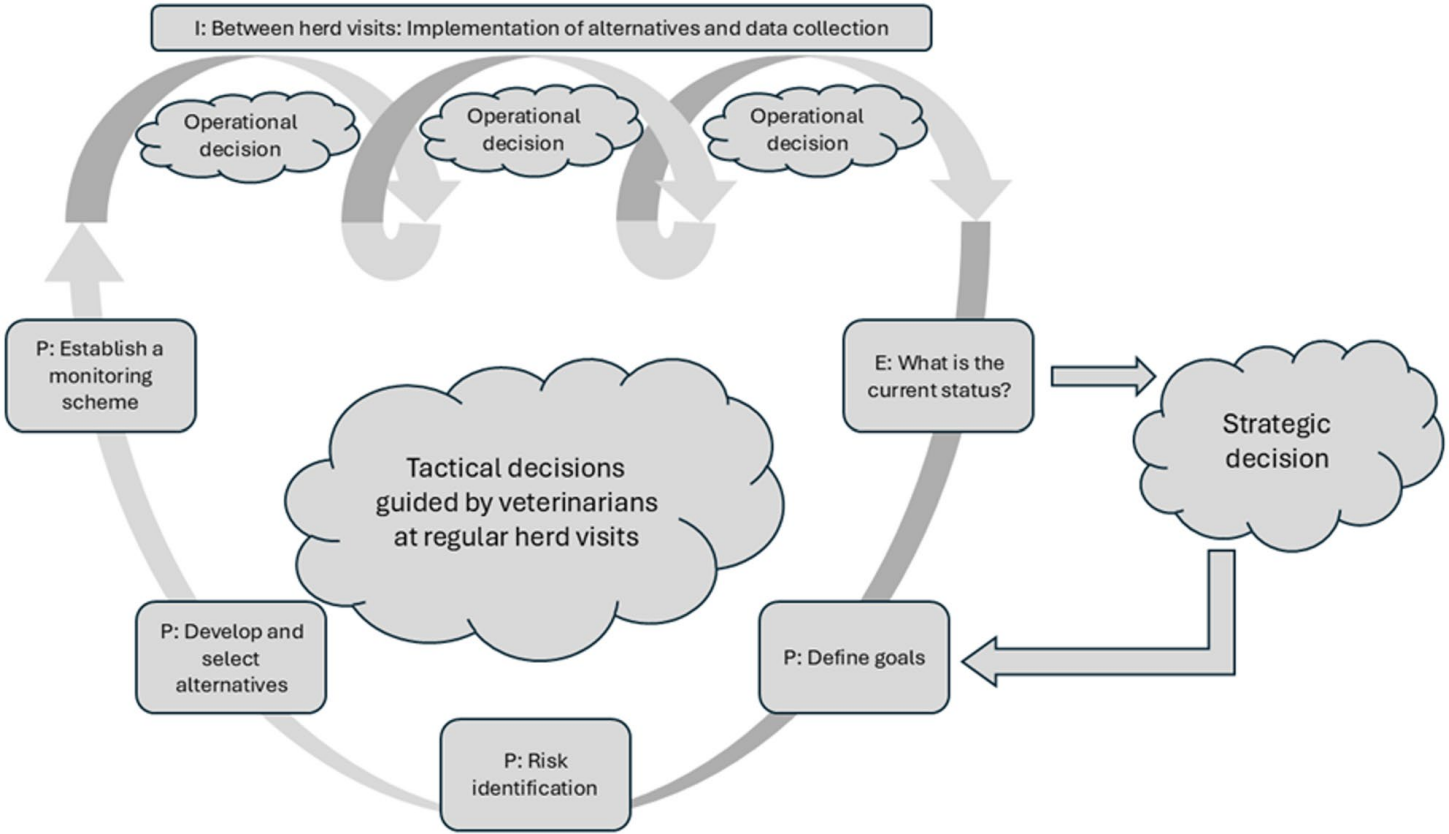
A model of the cyclic herd health management process (Inspired by Gray et al. [20, 22]; Gregory et al. [23]; Kristensen et al. [24]; Radostits [5]. P: Planning phase. I: Implementation phase, E: Evaluation phase)

The proposed theoretical framework is outlined in Fig. 2. In the sub-paragraphs below, each element of the tactical decisions is described in detail.

### Clarify current situation I

Herd health management was defined as a “continuous **cyclic process** dealing with planning, implementing, and **evaluating decisions** (…)”. Clarifying the current situation is an evaluation step, where previous initiatives are evaluated with reference to selected indicators of success relative to previously defined goals. This evaluation of specific efforts resulting from previous tactical decisions will be addressed in detail later in the paper.

In addition to the retrospective evaluations, new herd health problems may also be revealed in this stage. Defining new problems may happen at the livestock producer’s initiative, but livestock producers typically have high trust in their veterinarians and are open to discuss new topics on the veterinarian’s initiative [25, 26]. New problems may reveal themselves through:

- The observations, perceptions, or concerns of the herd personnel or livestock producer.
- Evaluation of routinely collected data monitoring health and production.
- The veterinarian’s herd audit.
- External factors like new legislation, regional herd health campaigns, changes in supply chains etc.

Furthermore, threats to the herd health or current practices that may pose a future problem can call for attention now. For instance, the potential introduction of an infectious disease to the herd can pose a threat to the herd health [25].

### Define goals

There is controversy in the literature on farm management about whether defining goals is something separate from the management process or embedded within it [20]. In the model proposed in Fig. 2, it is embedded in the process.

### Goals for health, productivity or externalities

The “goals” (“targets” in other terminologies) of herd health management are proposed to fall within three domains: health, productivity, and externalities.

Livestock producers often put a strong emphasis on health in their view of what animal welfare is [27]. Therefore, improved health can be motivated by a desire to keep animals with perceived good welfare. In line with this, good health can lead to increased work satisfaction, and bad health can be frustrating [28]. Even though herd health management seeks to promote herd health, the producer’s goal is often the expected benefits of enhanced health, e.g., improved growth or yield, or reduced costs for feed, medicine, or labour; that is, increased productivity.

### Defining goals

The S.M.A.R.T. model describes how management goals (in a broad sense) should be formulated, and supposedly, it also works in the present context. Goals should be Specific, Measurable, Assignable, Realistic, and Time-related [29].

Motivation and perceived behavioural control and capability influence whether changes in management are successfully implemented. Therefore, veterinarians should assist livestock producers in picking goals that motivate them and that they feel capable of achieving (as previously reviewed [30]). Unfortunately, evidence suggests that veterinarians providing veterinary herd health consultancy commonly fail to align expectations on goals. In 2007, only 50% of Dutch dairy producers reported that their veterinarian was both aware of their goals and sought to achieve them with the advice they provided [21]. The veterinarian often bears an implicit assumption that the producer primarily wants guidance in order to improve productivity; however, livestock producers (silently) expect veterinary herd health consultancy to focus on items such as prudent antimicrobial use, animal health, and animal welfare, and their goals are formed by other values than monetary gain [31, 32]. In the model summarized in Fig. 1, Panel B, the “sum” of these values was termed the “farmer’s utility function” [24]. This function describes the livestock producer’s welfare, which was suggested to be made up of the following elements: “monetary gain, leisure time, animal welfare, working conditions, environmental preservation, personal prestige, and product quality” [24]. Supplementing this proposal, livestock producers’ utility functions has also been shed light on an empirical basis. The scientific literature on factors that shaping the way livestock producers intend to use antimicrobials has been reviewed [14]. For this specific matter, livestock producers’ intentions were shaped by both intrinsic factors, such as social norms, emotions, experience, fear, and perception of risk, as well as the surrounding world, for instance, the livestock producers economic situation, the local culture defining common practices, legislation, media and society, and the consumers’ opinions [14].

As elaborated upon later in this paper, the veterinarian can actively seek to influence the livestock producer’s values, for instance by raising the producer’s awareness of a problem. That is, aligning expectations on goals should not imply uncritically conforming to the livestock producers’ goals; it rather means explicitly discussing the goals.

In summary, the veterinarian should explore what concerns the producer, try to shape this, and thereby help to define goals regarding productivity, health, and externalities that are specific, measurable, assignable, realistic, and time related.

### Risk identification

Now, the current status has been clarified and goals defined. The difference between the two (status and goals) may be termed a *“*herd health problem*”* [5]. As stated in the definition of herd health management, the purpose is to resolve the herd health problem, i.e., to make the performance meet the goals [5]. To achieve this, the first step is to perform risk identification (see Fig. 2), i.e., explore the possible causes of the herd health problem. This requires the veterinarian’s expertise because a profound understanding of causation is necessary. Table 1 outlines some common approaches to risk identification and, with a basis in the causation of post-weaning diarrhoea in pigs, examples of measurements and the risk factors they address [33].

**Table 1:** Various approaches to risk identification: post-weaning diarrhoea in pigs as an example

Risk identification approach	Example of measurement	Risks identified
Observing the animals	Prevalence survey of abnormal/unwarranted behaviours, e.g., belly nosing, aggression, etc.	Post-weaning stress
Interviewing the producer and herd personnel	Going through a checklist of biosecurity items	Biosecurity breaches
Auditing and inspecting the production, its facilities, and procedures	Check feeders, feeding space, and water sources	Poor availability of feed and water
Diagnostic investigations	Microbiologic laboratory diagnostics performed on diarrheic faecal samples	Intestinal pathogens
Measurements of environmental factors	Airflow and temperature in the pen	Draught and cold air
Measurements of feed	Analysis of the feed composition	High crude protein level
Study production data	Checking the mean days in nursing for sows	Low weaning age

It has been proposed to apply the Hazard Analysis Critical Control Points (HACCP) system in the context of herd health management [34, 35]. The risk identification is termed “hazard analysis” in this system, but the approaches will be the same as described in Table 1.

### Develop and select alternatives

At this point, the current status has been established, goals have been defined, and a risk identification has revealed the possible reasons for a herd health problem. The next stage of a tactical management decision is to develop and select alternative management practices to be implemented in order to mitigate the herd health problem and ultimately achieve the defined goals (Fig. 2).

Developing alternatives is linked directly to the risk identification step: now that “risk X” has been identified as an important cause of the present health problem, what can possibly be done to remove it? Likewise, the selection of one or more alternatives is done by considering the following: which of the developed alternatives is believed to remove “risk X” most efficiently at the lowest possible cost in terms of resources, labour time, negative externalities, and compliance with the livestock producer’s personal values and preferences? This description of the decision-making is abstract and broad aiming to cover any given situation. To facilitate understanding here is a very simple example the management of nursery pig health:

- Risk identified: The room temperature is lower than recommended.
- Develop alternatives: Increase straw provision or adjust the floor heating.
- Selected alternative: Adjusting floor heating is convenient and therefore selected.

The selection of alternatives can be very complicated, because the veterinarian essentially seeks to predict the outcomes of the alternative practices. This forecasting may solely rely on the veterinarians’ expert opinion, but the complexity of this makes guidance from decision-support tools presenting the likely outcomes highly relevant. This can, for instance, be: simple calculations of the expected effect on health or productivity and on the cost of interventions; decision-trees advising the best alternative given the circumstances of the decision; fact sheets or tables summarizing the scientific evidence of effects of management procedures; or software for simulation-based forecasting of health or economy [36].

### Level of prevention

When developing and selecting alternatives, a thing to consider is the level of prevention [37]. When the risk identification has established a plausible cause of a disease, prevention can be applied at different levels, termed “primary prevention” and “secondary prevention” [37]. In relation to infectious diseases, primary prevention seeks to remove the animal´s exposure to the infectious agent (e.g., cleaning and disinfection, Specific Pathogen Free (SPF) production systems, internal and external biosecurity). In contrast, secondary prevention seeks to prevent colonization with an infectious agent from developing into an infection with clinical disease (e.g., implementing a vaccination program, prophylactic antibiotic treatment, feed-additives believed to promote gastrointestinal eubiosis or immunological functions).

The adoption of the HACCP system into herd health management is founded on a focus on primary prevention and the monitoring of indicators of (un)successful primary prevention rather than the outcome.

### Establish a monitoring scheme

The final planning decisions to be made is the selection of indicators and how they should be monitored to evaluate the effect (see Fig. 2). It also is important to consider how to evaluate the effect (i.e., data analysis) when planning the monitoring scheme. An introduction to methods for effect evaluation is provided later in this paper.

Often, data for which a monitoring scheme has already been established, e.g., routinely collected production data, can serve as an effect measure.

For some goals, typically related to mitigation of threats to the herd health, no changes are expected if alternatives are successfully implemented. For instance, after successfully improving biosecurity measures to prevent an infectious agent of being introduced into the herd no changes are expected/hoped for. While some will opt for active surveillance by regular blood testing in such cases, a passive surveillance of clinical signs of the given disease may also be deemed sufficient, thus exemplifying that active collection of data is occasionally irrelevant.

Sometimes, it will be relevant to collect data on a new indicator of herd health. In this regard, one must consider the type of indicator and how it can be monitored. Furthermore, it should be decided whether to measure direct indicators of herd health or to measure the expected causes creating the effects on herd health. We elaborate on these two issues below.

### Herd health indicators

Indicators must be directly related to the defined goals, and preferably an indicator should be picked for each goal. The indicators should ideally be reliable (i.e., the measurements consistently lie close to the true value) and have high validity in measuring the herd health problem of interest. Table 2 provides an overview of the different types of indicators with a non-exhaustive listing of some examples for each type of indicator.

**Table 2:** Types of herd health indicators with examples

*Clinical indicators*
Prevalence of clinical signs (e.g., diarrhoea, lameness, depression) Coughing index Treatments incidence against specific disease Water consumption Feed consumption
*Productivity indicators*
Average daily weight gain Feed-conversion ratio Mortality rate
*Post-mortem indicators*
Systematic recordings of herd personnel’s suspected cause of death in piglets Necropsy surveys performed by the veterinarian Histological evaluation of tissue from a sample of animals Data collected by post-mortem inspections at the slaughter line
*Diagnostic testing*
qPCR analysis of sock and rope samples of faeces and saliva Culturing of faecal samples from a representative sample of animals Elisa-based pen-side test on faecal samples from a representative sample of animals Serological detection of antibodies in blood and rope samples PCR analysis of blood, tissue and rope samples for viruses Concentration of colostrum derived antibodies in the blood in a sample of animals Haptoglobin measurements in the blood in a sample of animals Antibody measurements in the blood in a sample of animals
*Environmental indicators*
Gas concentration (e.g., ammonia) in the pen Temperature in the pen Humidity in the pen
*Resource indicators*
Consumption of utensils (e.g. nitrile gloves, cannulas, or scalpels) Consumption of antimicrobials Consumption of non-steroid anti-inflammatory medication

Feed and water consumption can be measured in real-time and subjected to algorithms that recognize deviations from the norm, possibly representing emerging disease outbreaks [38]. This is just one example of technology-assisted monitoring, a field currently expanding at a fast pace. It must be expected that an ever-increasing number of indicators can be monitored and processed automatically with still more advanced sensors and algorithms in the future. The porcine cough index illustrates the development well: A coughing index for pigs was first described more than 30 years ago [39], and later validated [40, 41]. These applications of the index relied on a person standing by the side of the pen, counting the number of coughs and number of animals manually, and clocking the time on their wrist-band watch. Some years ago, a smartphone application was released, facilitating the manual counting by tapping the screen, and storing of the results electronically [42]. More recently, an electronic device hanging above the pen and continuously recording the sounds emitted from the pigs was made commercially available [43]. It has a built-in algorithm processing the sound recordings into coughing frequencies. The levels are reported by a light bulb indicator on the device as low coughing frequency (green light), intermediate increase in the coughing frequency (yellow light) or marked increase in the coughing frequency (red light). Detailed information is available on connected computer devices [43].

### Monitoring performance or causes?

Relevant indicators are not necessarily indicators of the herd health. It can also be relevant to measure indicators of compliance with the planned practices (“process measurements”) [44]. An example listed in Table 2 is measuring the consumption of single-use nitrile gloves and other utensils as a measure of compliance with planned biosecurity procedures.

In line with the above, it is relevant to distinguish between monitoring indicators of performance and causes of performance. Intuitively, it will make sense to monitor performance. If the goal motivating a change in management is an increased growth rate, it will make sense to monitor the growth rate. However, it may also be reasonable to monitor the causes of this outcome. Causes of improved growth rate may be absence of intestinal pathogens, changes in feed composition, stocking density etc. which may be monitored to aid the veterinarian in evaluating why growth rate was (not) improved.

The principle of monitoring primary or secondary causes is related to the philosophy of moving towards primary prevention and is a fundamental principle in the HACCP-based herd health management concepts.

### Implementation

The tactical decisions occurring at herd visits are evaluation and planning activities. In between the tactical planning decisions, implementation activities are carried out under the producers’ responsibility (Fig. 2). Yet, the veterinarian has a crucial role in facilitating the actual implementation of the alternatives [45]. Livestock producers have high trust in veterinarians regarding herd health decisions [21, 25, 46], but despite this, they don’t always implement their recommendations [47], and this can be rooted in a sub-optimal advisory approach [21, 25, 48, 49]. In recent years, methodologies from the social sciences have been adopted and provided prosperous and valuable insights on the determinants of whether selected alternatives are actually implemented (reviewed in [30]). Specific recommendations (see [30]) generally have four different targets, which are to enhance the producer’s: problem awareness and sense of responsibility; trust in the effectiveness of the selected alternatives; (perceived) ability to implement the selected alternatives; and perceived benefits of implementing the alternatives [30]. The recommendations are also relevant when convincing a producer to define certain goals.

### Clarify the current situation II

Herd health management was defined as a cyclic process. Accordingly, we are now back where we started, clarifying the current situation; or more specifically, we are at the beginning of a new management cycle at a follow-up herd visit (see Fig. 2). Here, the retrospective evaluation should consider two things:

- Has the plan been successfully implemented?
- Has the implemented plan resulted in the desired effects?

### Has the plan been implemented successfully?

When evaluating, even the best veterinarian and livestock producer will experience that alternatives have not been implemented as planned. Non-compliant behaviour is heterogeneous [21, 47], and therefore veterinarians should not make uninformed assumptions about the reasons for the lack of implementation. To identify the reason(s), veterinarians can structure their interrogation using a modified ADKAR^®^ scheme [50, 51]. This entails investigating whether the unsuccessful implementation is rooted in lack of (1) **A**wareness of the herd health problem the alternative should resolve; (2) **D**esire to achieve the potential gains of implementing the selected alternative; (3) **K**nowledge on how to implement the alternative; (4) **A**bility to implement the alternative; or (5) **R**einforcement due to a neutral or bad experience with the change. The encouragement and guidance of the producer and the herd personnel are then focused on the item appearing to cause the unsuccessful implementation of the selected alternative [50, 51]. The previously introduced recommendations [30] correspond with deficiencies possibly identified by the ADKAR^®^ scheme.

### Has the plan resulted in the desired effect?

When evaluating the effect of adopting new management practices, the veterinarian essentially seeks to establish evidence of a causal relationship between the implemented alternative and the monitored herd health indicator. This is an ambitious mission that may lead to fallacies. Simply to measure and compare the herd health indicator before and after the adoption of a new management practice does not allow for strong inferences. Because the production fluctuates over time, more than just the new management practice will have changed [52].

The herd-specific randomized trial aims to circumvent this problem of time-wise confounding. It is therefore the strongest possibly approach to establish the causal relationship. This approach resembles clinical trials as known from research: Tagged animals or groups of animals (e.g. pens) are allocated randomly to either receive the usual practice or the new management practice. A target herd health indicator is monitored in both groups. The effect of the new practice is the difference in this measure between the “new practice group” and the “usual practice group” [52].

This state-of-the-art method is, however, rarely applied in practical herd health consultancy. It requires a great deal of resources to conduct, and it is not always possible to randomly allocate the new practice to a fraction of the animals in the herd. Therefore, it has been suggested to adopt the principles of evolutionary operation (“EVOP”) from industrial process management into herd health management. This approach aims to mimic some features of the herd specific randomized trial with designs that are more feasible to carry out in an ongoing production of livestock [53, 54].

To the authors’ knowledge, the advanced methods introduced above are not widely applied in herd health management, and often the evaluation of effects relies on simple comparisons of descriptive summary statistics of the monitored herd health indicators before and after the adoption of a new management practice.

Advanced statistical approaches have been developed to allow for stronger inferences even when using the simple “before-after” design, for instance: process behaviour charts (also termed “XmR charts”) showing a time-series of a moving average of the monitored herd health indicator with upper and lower “natural process limits” [44]; and state space models, which are (typically) Bayesian models, combining a priori expectations with observed data to forecast future development in the herd health indicator [14, 44].

## Conclusions

Herd health management and related terms is used inconsistently in the scientific literature, and various definitions has been proposed. In this review, herd health management was defined as a continuous, cyclic process dealing with planning, implementing, and evaluating decisions aiming to promote herd health, i.e., normal biological function, including homeostasis and the absence of disease among the animals populating a given farm, in order to achieve the producer’s goals regarding productivity, animal health, and externalities. The paper proposed a theoretical framework describing how herd health management is practiced. Herd health management includes decisions at both strategic, tactical and operational level. The steps involved in tactical decisions are an evaluation of the current situation, defining goals, identifying risks and planning new practices to be implemented and a way to monitor indicators of success.

## Data Availability

Not applicable.

## Declarations

### Prior publication

The paper is an edited version of the Chap. 4 in the first author’s PhD thesis [55]. There are major textual resemblances and overlap in the intellectual content presented in the two works.
